# Chromosomal rDNA Distribution Patterns in Clonal *Cobitis* Triploid Hybrids (Teleostei, Cobitidae): Insights into Parental Genomic Contributions

**DOI:** 10.3390/genes16010068

**Published:** 2025-01-09

**Authors:** Alicja Boroń, Anna Grabowska, Olga Jablonska, Lech Kirtiklis, Sara Duda, Dorota Juchno

**Affiliations:** Department of Zoology, Faculty of Biology and Biotechnology, University of Warmia and Mazury in Olsztyn, 10-719 Olsztyn, Poland; alibo@uwm.edu.pl (A.B.); agrabowska@iimcb.gov.pl (A.G.); olga.jablonska@uwm.edu.pl (O.J.); sara.felinska@student.uwm.edu.pl (S.D.); juchno@uwm.edu.pl (D.J.)

**Keywords:** *Cobitis* species, triploid hybrids, *5S* and *45S* rDNA, hybridization, polyploidy, gynogenetic reproduction, cytogenetic markers, karyotype

## Abstract

**Background:** Interspecific hybridization between relative species *Cobitis taenia* (with a diploid genome designated as TT), *Cobitis elongatoides* (EE) and *Cobitis tanaitica* (NN) and the successive polyploidization with transitions from sexuality to asexuality experienced by triploid *Cobitis* hybrids likely influence their chromosomal rearrangements, including rearrangements of ribosomal DNA (rDNA) distribution patterns. Previously, we documented distinct karyotypic differences: *C. elongatoides* exhibited bi-armed chromosomes while *C. taenia* showed uni-armed chromosomes with rDNA-positive hybridization signals, respectively. **Methods:** In this study, fluorescence in situ hybridization (FISH) with *5S* rDNA and *28S* rDNA probes was used to analyze and compare chromosomal distribution patterns of rDNAs in clonally reproduced triploid *Cobitis* hybrids of different genomic constitutions ETT, ETN, EEN and EET (referred to using acronyms denoting the haploid genomes of their parent species), and their parental species. **Results:** *Cobitis* triploid hybrids exhibited intermediate karyotypes with ribosome synthesis sites on chromosomes inherited from both parents, showing no evidence of nucleolar dominance. The rDNA pattern derived from the *C. elongatoides* genome was more stable in the hybrids’ karyotypes. Two and one submetacentric chromosomes with co-localized rDNAs were effective markers to ascertain *C. elongatoides* diploid (EE) and haploid (E) genomes within the genome of triploid hybrids, respectively. Fewer *5S* rDNA loci were observed in diploid (TT) and haploid (T) chromosome sets from *C. taenia* in ETT and EET females. *C. taenia* and *C. tanaitica* exhibited similar rDNA distribution patterns. **Conclusions:** The karyotypes of triploid *Cobitis* hybrids reflect the genomic contributions of their parental species. Variability in rDNA distribution patterns suggests complex genomic interactions in *Cobitis* hybrids resulting from polyploidization and hybridization, potentially influencing their reproductive potential.

## 1. Introduction

Loaches of the genus *Cobitis* are small bottom-dwelling freshwater fish widely distributed in Europe that mainly form diploid–polyploid populations. They are good models for studying interspecific hybridization and polyploidization in evolution. Triploid hybrid *Cobitis* females, which dominate in Central Europe, arise from diploid hybrid clonal lineages formed by three parapatric species [1,2]; the most widely distributed species are *Cobitis taenia*, with a diploid karyotype 2n = 48 (with a diploid genome designated as TT), *Cobitis elongatoides* (2n = 50, EE) and its sister species *Cobitis tanaitica* (2n = 50, NN) [3,4,5,6]. Hybridization among these sexual species leads to the formation of diploid hybrid females of genetic constitution with 49 (ET) or 50 (EN) chromosomes [3,4,7,8]. Hybrid males are usually sterile due to abnormal chromosome pairing in meiosis. Diploid F1 hybrid females undergo genome endoreplication in a portion of their gonocytes just before meiosis, leading to the formation of clonal gametes [9,10]. Occasionally, their diploid eggs are incorporated by the sperm of the parental species, producing triploid gynogenetic *Cobitis* females [7,10,11,12,13]. Therefore, diploid and triploid *Cobitis* hybrids, reproducing only asexually by gynogenesis, deal with genome incompatibilities and bypass hybrid sterility. The induction of this asexual mode of reproduction requires the presence of sperm donors from males of the coexisting sexual species. In the majority of *Cobitis* populations in Poland, clonally reproducing triploid hybrid females coexist with sexual *C. taenia* or, less frequently, *C. elongatoides*, and a few tetraploid hybrids, which arise through the incorporation of the sperm nucleus into unreduced clonal eggs laid by triploid females [1,10,11,12,14]. The great morphological likeness between *Cobitis* hybrids and sexual species necessitates the use of cytogenetic or molecular methods to distinguish between them.

The parental species of *Cobitis* hybrids differ in their karyotypes. These karyotypes have diverged from a common ancestor by various chromosomal rearrangements involving one possible fusion and numerous pericentric inversions [7,15]. Concerning the time of origin, triploid *Cobitis* hybrid females, which occur naturally, are a mixture of ancient clonal lineages formed from diploid clonal lineages dating back at least to the last interglacial period and lineages of recent origin from the Holocene epoch [1,4,16]. Interestingly, regardless of the different ages of origin, both diploid and triploid clonal *Cobitis* hybrids exhibit a long-term karyotypic stability; neither differences in chromosome number nor in their morphology are observed. Their karyotypes contain sets of chromosomes derived from each of their respective parental species [7].

The karyotype structure of parental species, cytogenetic analyses using C-banding and rDNA location may be pre-used to indicate the possible contribution of the karyotype structures of parental species to the formation of *Cobitis* triploid hybrids [3,17,18,19,20]. Genotyping based on the assessment of allozyme loci [3,4,6], sequencing of multiple mitochondrial and nuclear loci, molecular markers like the S7 gene intron [1,4], species-specific microsatellite loci [5,21], the polymorphism of ISSR markers [22], polymorphic satellite markers [10,13,15] and genomic in situ hybridization (GISH) [7] have proven to be very successful methods for identifying the parental species in the genomes of *Cobitis* hybrids.

Several triploid *Cobitis* hybrids with different karyotypes (3n = 73; 3n = 74; 3n = 75), reflecting their various genomic constitutions, have been found in Central Europe [1,2,3,4,7,17,18]. In Poland, *C. taenia* and *C. elongatoides* are widespread throughout the Odra River basin, but they have never been found to co-occur [1,18]. Moreover, *C. elongatoides* has not been found within the less studied *Cobitis* populations inhabiting the Vistula River drainage [11,18,23]. In addition, the genome of *C. tanaitica* as a parental species of triploid *Cobitis* hybrids is inherited clonally, and this species has not been reported in Poland to date [7,11,15,18].

In higher eukaryotes, ribosomal DNAs (rDNAs) are present in multiple copies and are arranged in two families. The nucleolus major rDNA (*45S* rDNA) family codes for 5.8S, 18S and *28S* rRNA, which are located at nucleolus organizer regions (NORs), and the non-nucleolus minor (*5S* rDNA) family codes for *5S* rDNA [24,25]. Data about these rDNAs’ numbers and positions on chromosomes are important and have been intensively used as species-specific markers in taxonomy, animal breeding, phylogenetics and evolutionary biology, as probes derived from their conserved regions hybridize to chromosomes of divergent biological taxa [26,27,28,29,30,31,32].

Some data concerning the chromosomal distribution pattern of *45S* rDNA and/or *5S* rDNA have been collected in the following *Cobitis* species: *C. taenia* [8,19,33,34], *C. elongatoides* [8,19], *Cobitis vardarensis* [35] and *Cobitis strumicae* [36]. Less abundant, however, are data on the chromosomal location of rDNAs in diploid and/or polyploid *Cobitis* hybrids [8,18,19,33,37].

The clonal reproduction of triploid *Cobitis* hybrid females provides a unique opportunity to study the inheritance patters of heterozygous chromosomes in hybrid lineages [7,15]. Among other species, the chromosomal inheritance of diploid and allotetraploid hybrids from interspecific hybridization of the red crucian carp (*Carassius auratus* red var.) and the common carp (*Cyprinus carpio*) in successive generations was studied by applying *5S* rDNA fluorescence in situ hybridization (FISH) [38,39]. However, data on the chromosomal inheritance of parental rDNA distribution patterns in hybrids and/or polyploid fish taxa are relatively scarce [40,41,42,43,44,45]. Previously, we documented the chromosomal inheritance of the *5S* and *28S* rDNA distribution patterns in *C. taenia* and *C. elongatoides* and their wild-type and experimentally induced diploid hybrids of the TE and ET genome compositions [8]. We confirmed that both these parental species exhibit distinct chromosomal rDNA distribution patterns; the *5S* and *28S* rDNA-positive uni-armed and bi-armed chromosomes are characteristic for *C. taenia* and *C. elongatoides*, respectively [18,34].

The above-mentioned results [8] and those concerning the long-term stability of *Cobitis* hybrids’ karyotypes [7] inspired us to perform the study presented in this paper. In this study, we compare rDNA chromosomal distribution patterns in different clonally reproduced triploid *Cobitis* hybrids of ETT, ETN, EEN and EET genome constitutions and their parental species. Using FISH, we assessed whether nucleolar organizer regions (NORs) were inherited from all parental species or whether nucleolar dominance occurs in triploid hybrids. This phenomenon manifested, e.g., in the inheritance of the chromosomal distribution pattern of *45S* DNAs from one of the parents as, for example, has been described in tetraploid hybrid lineages including polyploids derived from the distant hybridization of *C. auratus* red var. and blunt snout bream *Megalobrama amblycephala* [43,45]. Additionally, we examined whether the number and location of *28S* and *5S* rRNA sites of triploid hybrids reflect parental patterns and whether a reduction in *28S* rDNA and *5S* rDNA occurs in triploid hybrids of different genome composition in comparison with their parents, as it was observed in diploid–polyploid *Squalius* complex [46] and diploid *Cobitis* hybrids [8]. As some allotriploid *Cobitis* genomes resulted from hybridization involving a third species, *C. tanaitica*, the presented results provide new insights into the cytogenetic features of this species.

## 2. Materials and Methods

This study focused on triploid hybrid females of *Cobitis* collected from diploid–tetraploid populations in the Pilica River, near Mysiakowiec village (51°34′29.6″ N, 20°20′16.8″ E; 20 individuals) and the Bug River, near Stary Bubel village (52°15′53.3″ N, 23°11′58.2″ E; 2 individuals); both rivers are part of the Vistula River basin. The fish were transported alive to the laboratory and immediately subjected to genotyping and karyotyping. Each individual was injected with 0.05% colchicine solution (Sigma-Aldrich, Saint Louis, MO, USA) at a dose of 0.1 mL per 10 g of fish weight and then placed in a well-aerated aquarium. After 60–70 min, the fish were sacrificed using an overdose of anesthetic (MS-222, Sigma-Aldrich, Saint Louis, MO, USA; 100 mg/L). Kidney samples for karyotyping as well as muscle and dorsal fin tissue samples were collected from the deceased fish for genotyping.

The parental subgenomes of these triploid hybrid females were determined by combining genotyping methods and karyotype analysis. The hybrid females studied were referred to using acronyms denoting the haploid genomes of their parent species: the Danube loach *C. elongatoides* (EE), the spined loach *C. taenia* (TT) and the species *C. tanaitica* (NN). The genotypes of the examined individuals were determined based on previously established and routinely applied molecular markers, including the first intron of the S7 gene [3] and selected species-specific microsatellite loci [5,21]. Total genomic DNA was extracted from either muscle or dorsal fin tissue using the Genomic Mini kit (A&A Biotechnology, Gdansk, Poland) according to the manufacturer’s protocol. The intron of the nuclear S7 gene was amplified following Janko et al. [3]. PCR products were purified using the Clean-Up AX kit (A&A Biotechnology, Gdansk, Poland) and then commercially sequenced by Genomed (Warsaw, Poland). The resulting sequences were manually edited and aligned using BioEdit [47]. Microsatellite fragment-length analyses were performed using an ABI PRISM 3130 Genetic Analyser sequencer (Applied Biosystems, Waltham, MA, USA) with GeneScan-500 LIZ (Thermo Fisher Scientific, Waltham, MA, USA) as the internal size standard. Alleles were manually scored using GeneMapper v. 3.7 software (Applied Biosystems).

The triploid hybrid females of *Cobitis* examined in the current study represented three groups of different chromosome numbers (3n = 73; 3n = 74; 3n = 75) and four groups of different genome constitutions (ETT, EEN, ETN, EET) according to the parental genomes.

### 2.1. Karyotype Analysis

Chromosome distributions were prepared from cephalic kidney cells using standard air-drying and splash techniques described by Ráb and Roth [48], with modifications [34]. The cell suspensions are currently stored at −20 °C at the Department of Zoology, University of Warmia and Mazury in Olsztyn, Poland. Giemsa staining was performed according to the standard procedure. Metaphase chromosomes were classified using the nomenclature proposed by Levan et al. [49] into four morphological categories: metacentrics (m), submetacentrics (sm), subtelocentrics (st) and acrocentrics (a). It was assumed that the first two categories (m, sm) are bi-armed chromosomes, while the others (st, a) are uni-armed. Chromosomes were counted in at least 12 metaphase plates per individual and analyzed using MultiScan v. 14.02 software (Computer Scanning Systems, Warsaw, Poland; multiscan.idsl.pl) with the Karyotype supplement.

### 2.2. Analysis of rDNA(s)

Fluorescence in situ hybridization was performed on a total of 307 metaphase plates from 22 triploid hybrid individuals following the procedure of Fujiwara et al. [50], with modifications by Boron et al. [34]. The *5S* rDNA and *28S* rDNA probes were amplified by PCR, as described by Kirtiklis et al. [51], and labelled by nick translation with biotin-16-dUTP (20-deoxyuridine 50-triphosphate, Roche, Mannheim, Germany; www.roche.com) and digoxigenin-11-dUTP (Roche, Mannheim, Germany), respectively. Chromosome slides were pre-treated with RNase and denatured with formamide before hybridization with 200 ng of rDNA probes per slide. Post-hybridization washing was performed under low stringency conditions (70% formamide, 37 °C, 20 min). Detection of *5S* and 28S rDNA signals was obtained using avidin-fluorescein isothiocyanate (FITC; Roche, Mannheim, Germany) and anti-digoxigenin-rhodamine (Roche, Mannheim, Germany), respectively. Slides were counterstained with 4,6-diamidino-2-phenylindole dihydrochloride (DAPI; Vector, Newark, CA, USA). Hybridization signals were observed in at least 12 metaphase plates per individual under a Nikon Eclipse 90i fluorescence microscope (Nikon, Tokyo, Japan; www.nikon.com) using the set of filters for DAPI, FITC and rhodamine. Dual-color FISH images were captured using a high-resolution ProgRes MFcool camera (Jenoptik, Jena, Germany; www.jenoptik.com) and processed with Lucia software v.2.0 (Laboratory Imaging, Prague, Czech Republic; www.lim.cz).

## 3. Results

### 3.1. Karyotypes

**Figure 1:**
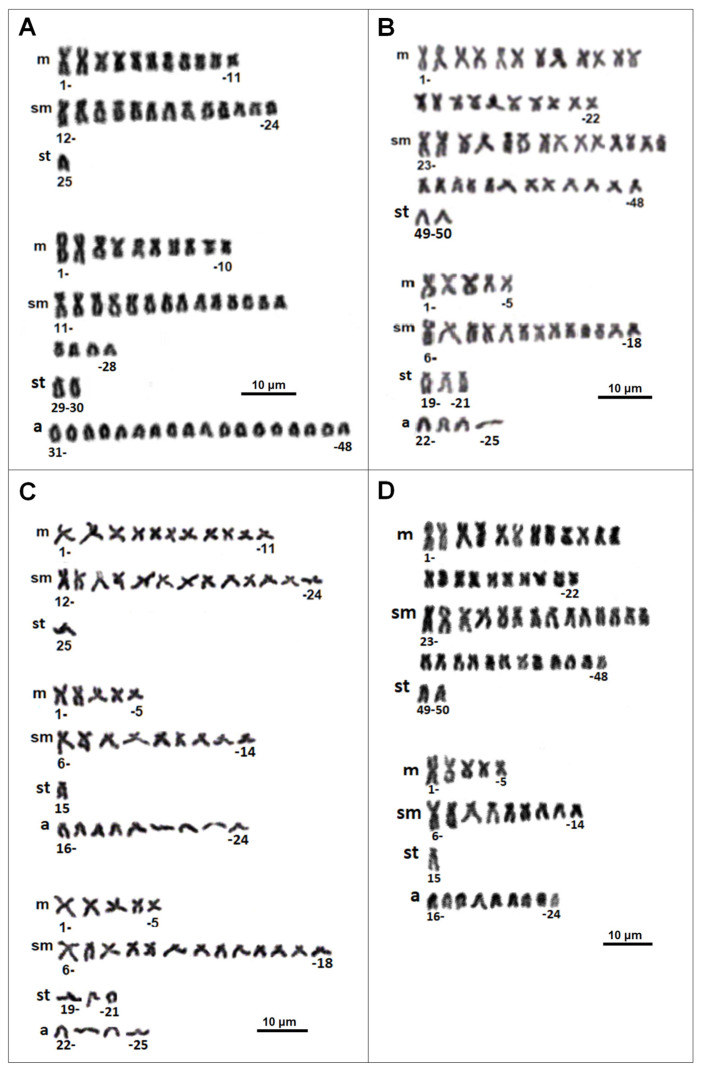
The karyotypes of triploid hybrid females of *Cobitis*. (**A**) Karyotype of female 3n = 73 (ETT) divided into chromosomes representing the parental species, *Cobitis elongatoides* (n = 25) and *Cobitis taenia* (2n = 48). (**B**) Karyotype of female 3n = 75 (EEN) divided into chromosomes representing the parental species, *C. elongatoides* (2n = 50) and *Cobitis tanaitica* (2n = 25). (**C**) Karyotype of female 3n = 74 (ETN) divided into chromosomes representing the parental species, *C. elongatoides* (n = 25), *C. taenia* (n = 24) and *C. tanaitica* (n = 25). (**D**) Karyotype of *Cobitis* female 3n = 74 * (EET) divided into chromosomes representing the parental species, *C. elongatoides* (2n = 50) and *C. taenia* (n = 24).

The complete metaphases of triploid hybrid females of *Cobitis* consistently displayed chromosome numbers corresponding to the expected combinations of the haploid parental genomes. Thus, ETT hybrids exhibited 3n = 73 chromosomes; ETN and EET hybrids displayed 3n = 74 chromosomes; and EEN hybrids demonstrated 3n = 75 chromosomes. A subset of metaphases from 22 hybrid females was arranged in a decreasing size order to demonstrate the consistency of the karyotype (Figure 1). The number of chromosomes and the morphological categories observed in all analyzed triploid hybrids were consistent with the expectations based on the combination of the parental species’ karyotypes (Figure 1 and Table 1).

Four ETT *Cobitis* females (two from the Pilica River and two from the Bug River) exhibited a triploid chromosome number of 3n = 73 and a karyotype composed of 21 metacentric, 31 submetacentric, 3 subtelocentric and 18 acrocentric chromosomes (NF = 125) (Figure 1A and Table 1). These hybrids were identified as possessing a haploid parental genome (E) of *C. elongatoides* (11 metacentrics, 13 submetacentrics and 1 subtelocentric) and a diploid genome (TT) of *C. taenia* (10 metacentrics, 18 submetacentrics, 2 subtelocentrics and 18 acrocentrics).

Four EEN *Cobitis* females exhibited a triploid chromosome number of 3n = 75. The karyotype was composed of 2n = 50 chromosomes, including 22 metacentric, 26 submetacentric and 2 subtelocentric derived from EE genome of *C. elongatoides*, and n = 25 chromosomes, comprising 5 metacentrics, 13 submetacentrics, 3 subtelocentrics and 4 acrocentrics, derived from the N genome of *C. tanaitica* (Figure 1B and Table 1).

The majority of the *Cobitis* hybrids analyzed (14 out of 22) possessed 3n = 74 chromosomes. Based on their genomic constitution, they could be classified into two groups: ETN and EET. The ETN females exhibited a karyotype comprising 21 metacentric, 35 submetacentric, 5 subtelocentric and 13 acrocentric chromosomes, with NF = 130 (Figure 1C and Table 1). The ETN genomic constitution was further distinguished by the presence of n = 25 chromosomes (11 metacentrics, 13 submetacentrics and 1 subtelocentric) derived from the E genome (*C. elongatoides*), n = 24 chromosomes (4 metacentrics, 9 submetacentrics, 1 subtelocentric and 9 acrocentrics) derived from the T genome (*C. taenia*) and the remaining n = 25 chromosomes (5 metacentrics, 13 submetacentrics, 3 subtelocentrics and 4 acrocentrics) derived from the N genome (*C. tanaitica*) (Figure 1C and Table 1).

Four triploid (3n = 74) *Cobitis* females, identified as EET, exhibited a diploid genome derived from *C. elongatoides* (EE) and a haploid genome derived from *C. taenia* (T). In these hybrids, chromosome sets representing the two parental species were observed: 2n = 50 chromosomes (22 metacentrics, 26 submetacentrics and 2 subtelocentrics) were derived from *C. elongatoides* (EE) and n = 24 chromosomes (comprising 5 metacentrics, 9 submetacentrics, 1 subtelocentric and 9 acrocentrics) were derived from the T genome of *C. taenia* (Figure 1D and Table 1).

### 3.2. rDNA Distribution Pattern

**Figure 2:**
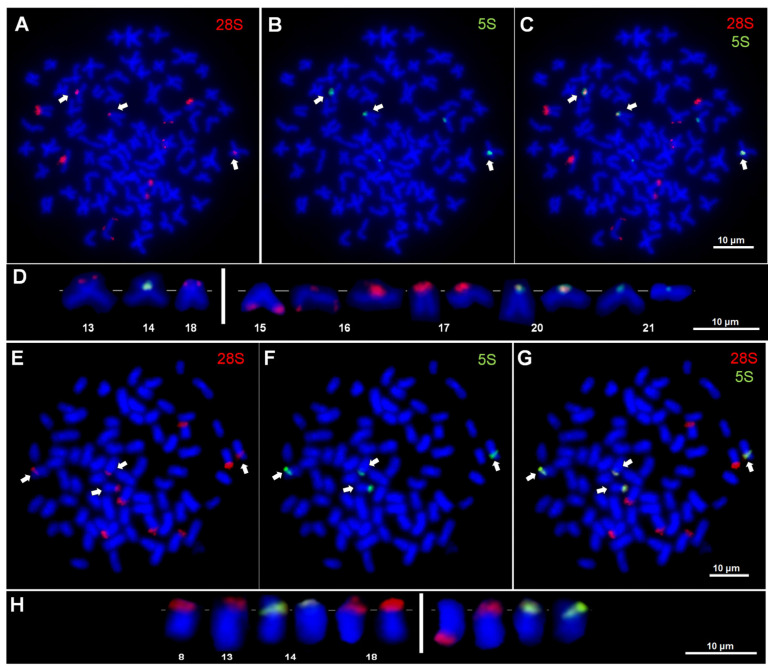
Double fluorescence in situ hybridization (FISH) with *28S* and *5S* rDNA probes: (**A**–**C**) Metaphase plate of hybrid *Cobitis* female (ETT, 3n = 73). (**D**) rDNA-bearing chromosomes, with numbering of chromosome pairs in the karyotype of *Cobitis elongatoides* (on the left) and *Cobitis taenia* (on the right) as parental species. (**E**–**G**) Metaphase plates of hybrid *Cobitis* female (EEN, 3n = 75). (**H**) rDNA-bearing chromosomes, with numbering of chromosome pairs in the karyotype of *C. elongatoides* (on the left) and unnumbered chromosomes of *Cobitis tanaitica* (on the right) as parental species. (**C**,**G**) Chromosomes with syntenic location of both *28S* and *5S* ribosomal genes; co-localizations of rDNA sites are indicated by arrows.

The triploid hybrid females (3n = 73) of ETT genomic constitution exhibited *28S* rRNA gene loci on ten or nine chromosomes in 89.7 and 10.3% of the 58 metaphase plates, respectively. The loci were mapped distally on the short arms of two differently sized submetacentrics (pairs 13 and 18 in the *C. elongatoides* karyotype) and proximally on the short arm of another submetacentric (pair 14 in the *C. elongatoides* karyotype). Furthermore, additional loci were observed in the pericentromeric position on six acrocentric chromosomes (pairs 16, 17 and 20 in the *C. taenia* karyotype) and distally on one subtelocentric chromosome (pair 15 in the *C. taenia* karyotype). In turn, one acrocentric chromosome (of the pair 16) exhibited *28S* rDNA hybridization signals in the pericentromeric region and also distally on the long arm (Figure 2A–D). Hybridization signals varied in intensity and were strong on four acrocentric chromosomes and weak on the remainder (Figure 2A,C,D). In metaphase plates with nine *28S* rDNA hybridization sites, one acrocentric chromosome lacked signals.

In ETT *Cobitis* females, a modal number of five *5S* rDNA hybridization sites (in 94.8% of 58 analyzed metaphase plates) was detected in the proximal region of the short arm of one submetacentric chromosome and in the pericentromeric regions of four acrocentric chromosomes. As with the *28S* rDNAs, the *5S* rDNA hybridization signals exhibited varying intensities and were categorized as stronger on the one submetacentric and three acrocentric chromosomes (pairs 20 and 21 in the TT karyotype) and weaker on the remaining acrocentric chromosome (pair 21) (Figure 2B–D). All ETT *Cobitis* females (3n = 73) exhibited a frequent syntenic location of both *28S* and *5S* ribosomal genes, that is, situated proximally on the short arm of a large submetacentric chromosome (pair 14 in the *C. elongatoides* karyotype) and in the pericentromeric regions of two acrocentric chromosomes (pair 20 in the *C. taenia* karyotype) (Figure 2C,D). No differences have been observed between females of EET genome constitution from the Bug and Pilica Rivers in the karyotypes and chromosomal distribution patterns of the rDNA sequences studied.

A modal number of ten *28S* rDNA hybridization sites was observed in 91.4% of 58 analyzed metaphase plates of the triploid hybrid (3n = 75) females of the EEN genomic constitution. The loci were situated in a proximal position on the short arms of two differently sized submetacentric chromosome and one metacentric chromosome, as well as on four uni-armed chromosomes (in the pericentromeric region of three acrocentrics and distally on the long arm of a subtelocentric chromosome) (Figure 2E,G,H). The fluorescence intensity of signals on one acrocentric and one submetacentric chromosome was consistently weaker than the others (Figure 2E,G,H).

Most frequently (in 96.6% of metaphase plates examined), four *5S* rDNA sites were observed in the chromosome sets of EEN females (Figure 2F–H). The loci were observed to be situated proximally in the short arm of two submetacentric chromosomes (pair 14 in the karyotype of *C. elongatoides*) and in the pericentromeric regions of two acrocentric chromosomes. The signals observed on two submetacentric chromosomes (pair 14) and one of the acrocentric chromosomes were stronger than the others (Figure 2F). In all specimens, four chromosomes with rDNA co-localization were observed: two submetacentrics and in the pericentromeric region of two acrocentrics.

**Figure 3:**
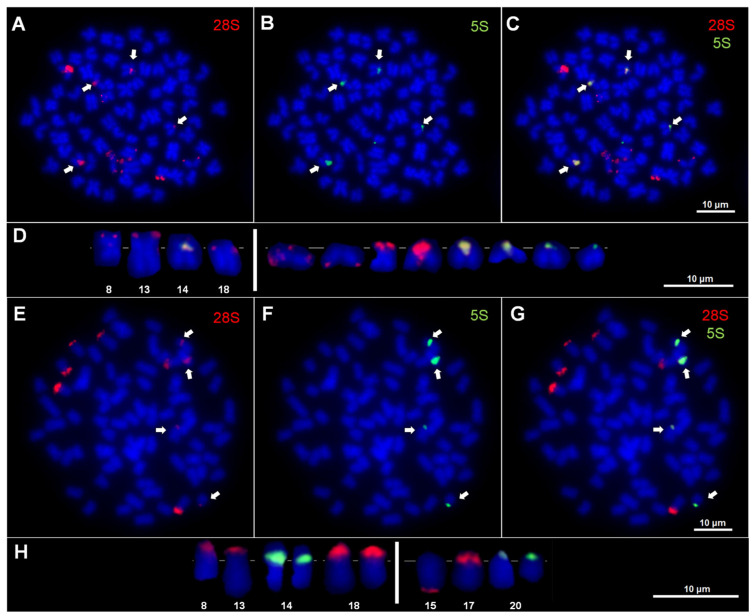
Double fluorescence in situ hybridization (FISH) with *28S* and *5S* rDNA probes: (**A**–**C**) Metaphase plate of hybrid *Cobitis* female (ETN, 3n = 74). (**D**) rDNA-bearing chromosomes, with numbering of chromosome pairs in the karyotype of *Cobitis elongatoides* (on the left) and unnumbered chromosomes of both *Cobitis taenia* and *Cobitis tanaitica* (on the right) as parental species. (**E**–**G**) Metaphase plates of *Cobitis* hybrid female (EET, 3n = 74 *). (**H**) rDNA-bearing chromosomes, with numbering of chromosome pairs in the karyotype of *C. elongatoides* (on the left) and *C. taenia* (on the right) as parental species. (**C**,**G**) Chromosomes with syntenic location of both *28S* and *5S* ribosomal genes; co-localizations of rDNA sites are indicated by arrows.

Triploid (3n = 74) *Cobitis* females of the ETN genomic constitution showed a modal number of eleven *28S* rDNA hybridization sites (observed in 93.2% out of 132 analyzed metaphase plates). These sites were located on the short arms of four bi-armed chromosomes (one small metacentric and three submetacentrics of different sizes) derived from the N genome of *C. elongatoides*, whereas seven others were in acrocentric chromosomes derived from both the T and N genomes of *C. taenia* and *C. tanaitica*, respectively (Figure 3A,C,D).

One acrocentric chromosome exhibited signals in the pericentromeric region and also distally on the long arm. Another acrocentric chromosome exhibited these signals solely distally on the long arm. The fluorescence intensity of signals varied among chromosomes, where two of them located in pericentromeric regions on acrocentrics and one on a submetacentric chromosome were always stronger than others (Figure 3A,C,D).

The modal number of five *5S* rDNA hybridization sites (detected in 95.5% of the 132 analyzed metaphase plates) was observed in triploid *Cobitis* females of ETN genome constitution. The sites were located proximally on the short arm of one submetacentric chromosome, a configuration typically observed in the E genome of *C. elongatoides* (n = 25 chromosomes) and in centromeric regions of four acrocentric chromosomes. As with the *28S* rDNAs, the *5S* rDNA hybridization signals exhibited variation in intensity and were classified as stronger both on the submetacentric chromosome (from pair 14 in the *C. elongatoides* karyotype) and two acrocentric chromosomes, and weaker on the others (Figure 3B–D). The co-localization of both analyzed rDNA sequences was observed in four chromosomes: one submetacentric and three acrocentrics (Figure 3C,D).

**Figure 4:**
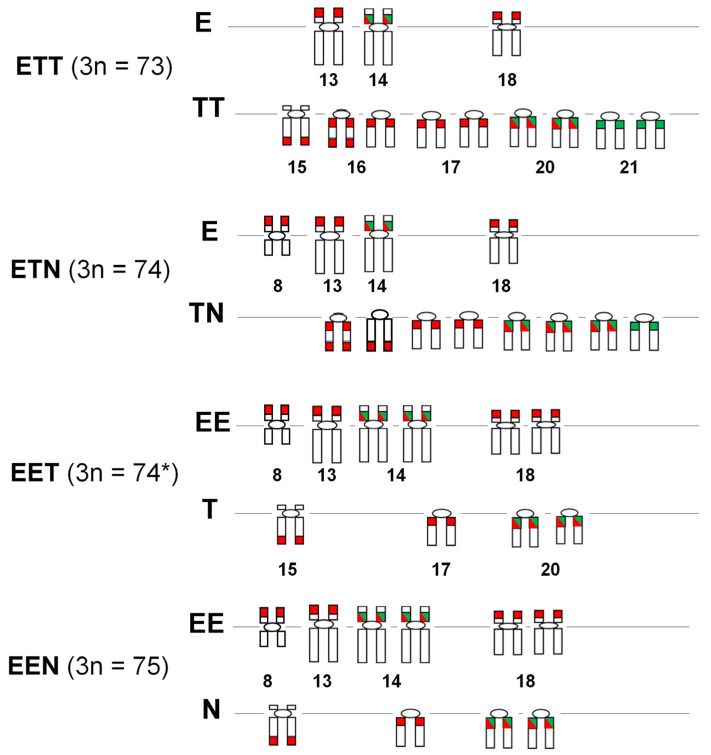
Schematic representation of the *5S* (green) and *28S* (red) rDNA distribution patterns in the karyotypes of triploid hybrid females of *Cobitis* of four different genome compositions (ETT, ETN, EET and EEN) divided into bi-armed chromosomes characteristic in the *Cobitis elongatoides* karyotype, uni-armed chromosomes characteristic in the *Cobitis taenia* karyotype (with numbering of chromosome pairs in the karyotype of these both parental species according to Grabowska et al. [8]) and unnumbered uni-armed chromosomes in the *Cobitis tanaitica* (N) karyotype.

Triploid (3n = 74 *) *Cobitis* females of EET genomic constitution showed a modal number of ten *28S* rDNA hybridization sites (in 94.9% out of 59 analyzed metaphase plates). The sites were situated on the short arms of six bi-armed chromosomes (one small metacentric and five submetacentrics) derived from the E genome of *C. elongatoides*, while the other four were located in the acrocentric chromosomes derived from the T genome of *C. taenia* (Figure 3E,G,H and Figure 4). The fluorescence intensity of the signals varied among the chromosomes, with those on the bi-armed chromosomes and in the pericentromeric region on one acrocentric chromosome displaying stronger signals than the others (Figure 3E,G,H).

The modal number of four *5S* rDNA hybridization sites (in 96.6% of 59 analyzed metaphase plates) was detected in triploid (3n = 74 *) *Cobitis* females of EET genome constitution. The hybridization sites were located in a proximal position on the short arms of two submetacentric chromosomes, exhibiting a pattern similar to that observed in the EE genome of *C. elongatoides* and in the centromeric regions of two acrocentric chromosomes, as seen in the T genome of *C. taenia* (Figure 3F–H). As with the *28S* rDNAs, the hybridization signals exhibited varying intensities and were classified as stronger on two submetacentrics (pair 14 in the *C. elongatoides* karyotype) and weaker on the remaining chromosomes (Figure 3F–H). The co-localization of both analyzed rDNA sequences was observed in four chromosomes: two submetacentrics and two acrocentrics (Figure 3G,H).

## 4. Discussion

The karyotypes of the analyzed ETT (3n = 73), ETN (3n = 74), EET (3n = 74 *) and EEN (3n = 75) triploid *Cobitis* hybrid females reflect the chromosome sets of their parental species, which is consistent with previous studies [3,4,7,15,18]. However, karyotyping alone cannot definitively determine the genomic composition of *Cobitis* hybrids. Nevertheless, our findings support the idea that chromosome number is an important criterion for verifying the genotypes containing *C. taenia* and *C. tanaitica* genomes as the parental species [3,4,16].

The chromosomal distribution patterns of *5S* and *28S* rDNAs in the triploid hybrid females were highly reproducible among individuals within the genome-constituted groups, providing this cytogenetic feature valuable for comparative analysis between allotriploid *Cobitis* females and their parental species. Notably, *C. elongatoides* exhibited both these rDNAs located on bi-armed and meta- and submetacentric chromosomes, while in *C. taenia*, they are located on uni-armed and subtelo- and acrocentric chromosomes [8,19,34]. This distinction allowed us to identify the parental origin of chromosomes carrying these sequences in the karyotypes of the triploid hybrid females analyzed in the current study. For instance, in the karyotype of EEN females, some uni-armed chromosomes with rDNA loci corresponded to the haploid set of *C. tanaitica*. Due to the similarities in the chromosomal distribution patterns of the studied rDNAs in the *C. taenia* and *C. tanaitica*, the genomic contribution of these species (TN) to ETN female hybrids were reported together (Figure 3A–C and Figure 4).

### 4.1. Karyotype Structure

Two groups of females, ETN (3n = 74) and EET (3n = 74 *), contain the ET genome along with the genome of *C. tanaitica* (N) and *C. elongatoides* (E), respectively. The karyotypes of these females differ because the karyotype of *C. tanaitica* (2n = 50, NN) contains more uni-armed chromosomes, either 10 [1,13] or 14 [7,15], this study compared to the karyotype of *C. elongatoides* (EE), which has only two uni-armed chromosomes. So, the karyotype of *C. tanaitica* adopted in the present study comprises 10 metacentric, 26 submetacentric and 14 subtelo- and acrocentrics chromosomes. These differences, along with our karyotype analyses, support the hypothesis that the genome of *C. tanaitica* likely originated from an initial *elongatoides*–*tanaitica* hybridization event that occurred in the pre-Holocene period [16]. This was followed by triploidization, which led to the formation of ETN hybrids. Available data further suggest an allochthonous origin of both ETN and EEN clones [1,5]. The taxonomy of some *Cobitis* species, including *C. tanaitica* is still being discussed [2,22].

Triploid *Cobitis* females (3n = 74) with varied karyotypes were previously detected by chromosome banding in diploid–polyploid populations in the Vistula River basin [18] and the Odra River in Poland [17]. The triploid hybrids described in this study, namely ETT (3n = 73), ETN (3n = 74), EET (3n = 74 *) and EEN (3n = 75), have also been observed in several populations across Central Europe, including the Odra River basin [1,3,7,18]. Among *Cobitis* triploids distributed in the Odra River basin and in samples from the Pilica River analyzed in the current study, genotyping indicated that most of them possess the ETN genome [1].

### 4.2. rDNAs Distribution Pattern

The triploid *Cobitis* hybrids analyzed in the current study, along with their parental species, *C. taenia* and *C. tanaitica*, exhibit multiple chromosomal localization of *5S* and *45S* rDNAs, with some regions showing co-localization [8,18,34]. In contrast, *C. elongatoides* and its relative, *C. vardarensis* [35], also possess multiple chromosomal localization of *45S* rDNAs [8]. Interestingly, among the triploid hybrid *Cobitis* females examined and their parental species, the number of *28S* rDNA hybridization sites was greater than that of *5S* rDNA (Figure 4). Additionally, results indicate that the number of *5S* rDNA sites in both triploid and diploid hybrids [8] were fewer compared to their parental species. These findings suggest that while the number and location of *5S* and *45S* rDNA loci are species specific, polyploids do not necessarily have a greater number of rDNAs hybridization sites [27,28,52]. For instance, some diploid species within the related genus *Botia* may have a greater number of *45S* rDNA clusters compared to some tetraploid species, which showed only two *45S* rDNA loci [29]. However, unlike in *Cobitis*, both diploids and tetraploids of the *Botia* genus generally exhibit a greater number of *5S* rDNA hybridization sites compared to *45S* rDNA, and this reduction in site number may reflect the gradual processes leading to the elimination of excessive rDNA clusters in polyploids [29]. Interestingly, the related cyprinids Cyprinidae exhibited greater variation in the number and chromosomal locations of *5S* rDNA sites compared to the more stable 18S rDNA distribution pattern [31].

According to our previous data [8], six *28S* rDNA hybridization sites were observed in the karyotypes of both female and male *C. elongatoides*. Three of these sites were located terminally on the short arm of the metacentric chromosome (pair 8) and proximally on the short arm of two submetacentric chromosomes (pair 14) in individuals of both sexes. The remaining three *28S* rDNA hybridization sites in the karyotype of female *C. elongatoides* were located terminally on the short arm of two large chromosomes (pair 13) and one medium-sized submetacentric chromosome (pair 18). In contrast, in the karyotype of males, these sites were observed on one large chromosome (from pair 13) and two medium-sized submetacentric chromosomes (pair 18). Two submetacentric chromosomes (pair 14) were species specific, containing both *28S* rDNA and *5S* rDNA sites located proximally on the short arm [8,19]. The karyotypes of diploid F1 hybrid males (TE) and females (ET) contained four and three *C. elongatoides* chromosomes with *28S* rDNA sites, respectively, including one chromosome from pair 14 (Figure 4). Conversely, in the karyotypes of *Cobitis* EET (3n = 74 *) and EEN (3n = 75), the diploid *C. elongatoides* genome (EE) was consistently represented by six bi-armed chromosomes with typical *28S* and *5S* rDNA hybridization sites, including two submetacentrics chromosomes (pair 14) containing both rDNAs. Significantly, the double set of EE chromosomes in EET and EEN female karyotypes reflects the rDNA distribution pattern found in *C. elongatoides* males [8]. This finding supports the hypothesis that triploid EET and EEN females originated from double hybridization, first producing ET or EN hybrids, and then followed by the fertilization of diploid eggs of these hybrids with sperm from *C. elongatoides* males. This suggests that the rDNA distribution pattern of *C. elongatoides* males seems to be conserved in the diploid EE genome of triploid females with EET genome composition.

In the haploid *C. elongatoides* genome (E), there were three chromosomes (one metacentric and two submetacentric) with rDNA loci in the karyotype of ETT (3n = 73) hybrid females, while the karyotype of ETN (3n = 74) hybrid females contained four (one metacentric and three submetacentric) chromosomes with rDNA loci (Figure 4). Interestingly, the same rDNA distribution pattern was observed in the E genome of diploid hybrid ET females and TE males, respectively [8]. As mentioned above, diploid *Cobitis* hybrids possess a single species-specific chromosome (from pair 14) with syntenic localization of both rDNAs. Consequently, the rDNA distribution pattern in the haploid *C. elongatoides* chromosome set of triploid hybrid *Cobitis* females ETT (3n = 73) and ETN (3n = 74) matched that of diploid hybrids, ET females and TE males, respectively.

The rDNA distribution pattern in the chromosomes of the studied triploid *Cobitis* females appears to correlate with the number of *C. elongatoides* genomes present in the hybrids. A defining characteristic of the karyotypes of *C. elongatoides*, diploid hybrids (TE and ET) and triploid females is the presence of one (in ETT and ETN females) or two (in EET and EEN females) submetacentric chromosomes (pair 14) with co-localization of both rDNA sequences proximally on the short arm. The number of these chromosomes reflects the number of haploid *C. elongatoides* genomes present in the triploid genomes of *Cobitis* females.

The karyotype of the second parental species, *C. taenia*, was characterized by nine and six uni-armed chromosomes with *28S* and *5S* rDNA sites, respectively, displaying slight differences between the sexes [8]. A subtelocentric chromosome (from pair 15) with *28S* rDNA located on the short arm, observed in the diploid female karyotype of *C. taenia*, was also present in F1 diploid hybrid ET females. However, such a chromosome was absent in the triploid *Cobitis* hybrids (ETT, ETN, EET) containing the T genome described in the current study (Figure 4). In turn, a chromosome of the same category (from pair 15) with *28S* rDNA site on the long arm, characteristic of both in karyotype of *C. taenia* females and males, was observed in TE diploid hybrid males. This chromosome appeared in the TT genome of ETT hybrid females, the haploid T genome of EET hybrid females and in the indirectly obtained N pattern of the EEN genome. Additionally, the “double-sided” acrocentric chromosome (pair 16) with a double-NOR site, previously identified as specific to the *C. taenia* karyotype [8,18,37], was exclusively observed in the TT and TN genome parts of hybrid females with ETT and ETN genome composition, respectively.

Interestingly, among the chromosomes with syntenic rDNA sites in the *C. taenia* karyotype, two chromosomes (pair 20) were found in females and four (pairs 19 and 20) in males. However, only one pair (number 20) was observed in the TT component of the ETT genome and the T component of the EET genome (Figure 4). Consequently, in the TT part within the ETT female karyotype, there were fewer chromosomes carrying NORs (seven) compared to the diploid karyotype of *C. taenia*, which has nine such chromosomes. Similarly, there was a reduced number of chromosomes (pairs 20 and 21) with *5S* rDNA sites or co-localized rDNA sites, with only two out of four of such chromosomes compared to *C. taenia* [8]. The hybridization process that led to the emergence of females with the ETT genome constitution likely involved two stages: initial hybridization resulting in the formation of diploid ET hybrids, whose diploid eggs were occasionally fertilized by *C. taenia* males, resulting in triploid clonal gynogenetic offspring.

When compared to diploid hybrid ET females, triploid females with the ETT genome exhibited three *C. elongatoides* chromosomes with rDNAs hybridization sites and nine *C. taenia* (TT) chromosomes. In the karyotype of the diploid ET female (2n = 49), the T genome was represented by six chromosomes, two of which (subtelocentric and acrocentric) contained *28S* rDNA sites located on the short arm and distally at the end of the long arm, respectively; these were not found in the ETT female karyotype. These comparisons indicate that the haploid (T) chromosome set of *C. taenia* present in the karyotypes of diploid (TE, ET) and triploid (EET) hybrids shows a more differentiated rDNA distribution pattern and a lower number of rDNA-bearing chromosomes than the diploid chromosome set of this species (TT) in the karyotypes of triploid hybrids (ETT).

The data indirectly revealed a previously unknown rDNA distribution pattern in the *C. tanaitica* karyotype, which appears very similar to that of *C. taenia*. In the karyotype of EEN females, the haploid genome (N) of *C. tanaitica* is represented by four uni-armed chromosomes with *28S* rDNA: one subtelocentric chromosome with a terminal location of the rDNA site and three acrocentric chromosomes with pericentromeric rDNA sites. Two of these acrocentric chromosomes also showed synteny with *5S* rDNA. This similarity in rDNA distribution patterns between *C. tanaitica* and *C. taenia* was also observed in the metaphases of female *Cobitis* ETN hybrids stained by FISH. Among the uni-armed chromosomes corresponding to the TN genomes, seven contained *28S* rDNA and four had *5S* rDNA, including three chromosomes with observed synteny with both rDNAs. Notably, one of these chromosomes corresponds to the ‘double-sided’ chromosome (pair 16) typical of the *C. taenia* karyotype, while another showed *28S* rDNA signals terminally located on the long arm, identified previously only in diploid ET hybrid females [8] (Figure 4). The observed similarity in the distribution of these rDNA sequences in the chromosomes corresponding to the T and N genomes of EET and EEN females, respectively, aligns with the previously reported cytogenetic and genetic similarities between *C. taenia* and *C. tanaitica* [1,4,6,19].

Notably, nucleolar dominance was absent in the clonal triploid *Cobitis* hybrids analyzed in this study, which were characterized by the intermediate karyotype with ribosomal synthesis sites on chromosomes inherited from two (ETT, EET and EEN) or three (ETN) parents. The observed numbers of both *28S* and *5S* rDNAs sites in the karyotypes of triploid *Cobitis* hybrid females were disproportionally inherited from their parental species. Similarly, in both wild-type and induced F1 diploid *Cobitis* hybrids (ET and TE) [8], the *28S* and *5S* rDNA signals were also inherited in a manner inconsistent with Mendelian ratios. The presence of nucleolar dominance, where one rDNA locus is silenced while the other remains active [43], was reported in other hybrids such as in allodiploid lineages of fish formed by distant hybridization of *M. amblycephala* and *Culter alburnus* [53], as well as in tetraploid hybrid lineages resulting from the hybridization of *C. auratus* red var. and *M. amblycephala* [43,45]. The lack of nucleolar dominance observed in both diploid and triploid *Cobitis* hybrids may be due to their gynogenetic reproduction and the subsequent absence of recombination, which could prevent the stabilization of nucleolar dominance in these hybrids. However, only a minor fraction of the hybrid’s gonocytes were able to duplicate their genomes due to significant genome incompatibilities [10]. Additionally, the absence of nucleolar dominance could indicate the hybrid’s greater resistance to genomic diversity from the parental species, a typical feature of clonal hybrids. So, from other sites, this process may reduce the reproductive potential of *Cobitis* hybrids.

In metaphases of ETT (3n = 73) and EET (3n = 74 *) triploid hybrids, corresponding to TT and T genomes from diploid and haploid chromosome sets, respectively, there was a lower number of chromosomes with co-localized *45S* and *5S* rDNAs compared to *C. taenia*. The chromosomal locations of *5S* rDNAs may vary among polyploid hybrids and their parental species [54], making *5S* rDNAs useful for analyzing hereditary relationships among them [39]. So, in the haploid T genome, EET females (3n = 74 *) and diploid ET hybrids exhibited only two *5S* rDNA loci (this study [8]). A similar reduction in *5S* rRNA gene loci observed in autotetraploid hybrids of *C. auratus* red var. and *M. amblycephala* seems to affect the formation of multivalents, interfering with meiosis, and potentially reducing reproductive barriers [41,42]. Conversely, the allotetraploid genomes of hybrids from these taxa have experienced genetic variations that most likely promote the overcoming of parental genome incompatibility [42]. Therefore, the genetic changes occurring in autopolyploids at chromosomal *5S* and *28S* gene loci appear to differ from those in allopolyploid genomes.

The observed differences in FISH signals intensity across the chromosomes of all analyzed triploid hybrid females were inconsistent among the parental species, revealing no clear pattern. In contrast, the diploid hybrids inherited two strong *5S* rDNA signals from *C. auratus* red var. and two weak signals from *C. carpio*, with one signal from each parent. Additionally, the strong FISH signals detected in induced allotetraploid hybrids were likely maternally inherited [38,39]. These observations highlight the variability in rDNA organization and transmission, which is attributed to the dynamic nature of these sequences [55,56].

The dynamic properties of rDNA contribute to their variability in location and copy number, often visible as differences in signal size. Such dynamics can cause chromosomal incompatibilities in hybrids, potentially leading to unbalanced gametes or disruption in meiosis. For example, the number of NORs, sites for ribosome synthesis on chromosomes containing *28S* rDNA, may decrease in hybrid progeny compared to their parents [46]. Alternatively, these NORs may be inherited from both parents [53]. This variability is a hallmark of hybrid genomes and reflects broader processes shaping hybrid evolution.

Biological processes such as interspecies hybridization linked with subsequent polyploidization, asexual reproduction, and infrequent meiosis, as observed in the evolution of polyploid *Cobitis* hybrids, contribute to rDNA heterogeneity [25]. In turn, mechanisms such as meiotic recombination, gene conversion, and loci elimination, may reduce rDNA heterogeneity [25]. Fewer rDNA loci observed in the chromosomes of triploid hybrid females of *Cobitis* compared to their parental species, as reported in this study, align with well-documented patterns of rDNA loci elimination (both *5S* and *45S*) in various polyploid plants and animals [25], including other fish taxa [46,57].

The number of chromosomal sites and/or rDNA loci directly influences rDNA homogeneity. Species with fewer rDNA loci are more likely to exhibit complete homogenization of rDNA units than those with more loci [25]. The positional context of individual rDNA units within the array also plays a role in their likelihood of undergoing homogenization. For instance, edge-located copies within rDNA clusters often evade homogenization [58]. On the other hand, rDNA clusters in telomeric and centromeric positions are more prone to homogenization. An example of this is observed in Northern pike *Esox lucius*, where highly amplified and homogenized *5S* rDNA are found at the pericentromeric positions of most uni-armed chromosomes [59].

The chromosomal localization of rDNAs in triploid *Cobitis* hybrids, reported in this study, primarily in pericentromeric and terminal regions, suggests the evolutionary stability of these regions compared to subtelomeric and interstitial ones. This stability highlights their potential role in maintaining rDNA functionality despite hybridization and polyploidization.

Cases where hybrids inherit distinct and recognizable rDNA patterns from both progenitors may indicate independent inheritance of parental rDNAs without homologous recombination and gene conversion [58]. This independent inheritance likely contributes to the observed variability in rDNA patterns and underscores the complex interplay between inheritance mechanisms and the evolutionary processes shaping hybrid genomes.

The rDNA sequences derived from the *C. elongatoides* genome exhibited greater stability and less variability in the karyotypes of all tested hybrids with ETT, ETN, EET and EEN genomic constitutions. Particularly, *5S* rDNA-bearing chromosomes have been consistently passed down through successive generations of hybrid fish lineages, including allodiploid and allotetraploid hybrids [38]. However, sequencing data indicate different hereditary characteristics of paternal-specific *5S* rDNA in diploid, triploid and tetraploid hybrids of *C. auratus* red var. × *Erythroculter ilishaeformis* [44]. In contrast to the more diverse distribution patterns of rDNA sequences in the haploid (T) or diploid (TT) chromosome sets of *C. taenia*, the triploid *Cobitis* hybrids with an EET genomic constitution exhibited a distinct pattern. Given that the *Cobitis* females under study are clonal hybrids and the offspring of gynogenetic triploid females, this mode of inheritance does not lead to different patterns of rDNA distribution compared to those observed in the parental species. No additional rDNA hybridization sites were identified in the hybrids beyond those observed in the karyotypes of the parental species, *C. taenia* and *C. elongatoides*. The striking similarity in rDNAs distribution observed in this study in the karyotypes of *C. taenia* and *C. tanaitica* is consistent with the findings of other researchers who have noted the striking similarities in the karyotypes of both species [7]. These findings indicate that it is not possible to identify *C. taenia* or *C. tanaitica* as the parental species of clonal triploid *Cobitis* females based solely on the chromosomal location of the *5S* and *28S* rDNA sequences, due to their similarities in both species. However, karyotype analysis serves as an effective complement to genotyping for determining the presence of these species in the genomes of triploid *Cobitis* hybrids.

## 5. Conclusions

Our results enhance the understanding of the chromosomal distribution patterns of *5S* and *45S* rDNA sequences in clonal triploid hybrid females of *Cobitis* with various genome compositions, formed by hybridization between relative species (*C. taenia*, *C. elongatoides* and *C. tanaitica*) and successive polyploidization. We documented the following findings: (a) the analyzed *Cobitis* hybrids possess chromosome sets derived from relevant parental species; (b) their karyotypes, stained with FISH using *5S* and *28S* rDNA as probes, exhibited bi-armed and uni-armed chromosomes with hybridization signals characteristic of *C. elongatoides* and *C. taenia*, respectively; (c) some uni-armed chromosomes with *5S* and *28S* rDNA sites in EEN triploid hybrids may correspond to the *C. tanaitica* karyotype; (d) one or two submetacentric chromosomes with both analyzed rDNAs in synteny on the short arms may aid in the identification of *C. elongatoides* as haploid or diploid genomes, respectively; and (e) the chromosome sets of *C. taenia* in triploid hybrids contained fewer chromosomes with *5S* rDNA loci. The karyotypes of triploid *Cobitis* hybrids reflect the genomic contributions of their parental species. The variability in rDNA distribution patterns suggests complex genomic interactions in *Cobitis* hybrids resulting from polyploidization and hybridization that may influence their reproductive mechanisms. These findings provide insights into the evolutionary biology, cytogenetics and taxonomy of hybrids, emphasizing the role of rDNA distribution as a marker for understanding hybridization and polyploidization.

## Figures and Tables

**Table 1:** Karyotype characteristics and genomic constitution of analyzed clonal triploid hybrid females of *Cobitis* (the current paper) and their parental species [3,7,8,34]. Sets of haploid genomes: (E)—*Cobitis elongatoides*; (T)—*Cobitis taenia*; (N)—*Cobitis tanaitica*. Chromosome categories: m—metacentric; sm—submetacentric; st—subtelocentric; a—acrocentric. Symbol: NF—chromosome arm number.

Ploidy Level and Genome Composition		Number of Chromosome Categories				NF
		m	sm	st	a	
Parental species						
*C. elongatoides* (EE)	2n = 50	22	26	2	-	98
*C. taenia* (TT)	2n = 48	10	18	2	18	76
*C. tanaitica* (NN)	2n = 50	10	26	6	8	86
*Cobitis* hybrid females						
ETT	3n = 73	21	31	3	18	125
ETN	3n = 74	21	35	5	13	130
EET	3n = 74 *	27	35	3	9	136
EEN	3n = 75	27	39	5	4	141

(*) distinguish one of two karyotypes with the same number of chromosomes.

## Data Availability

The original contributions presented in the study are included in the article, further inquiries can be directed to the corresponding author.
